# Hierarchical two-step floating catchment area (2SFCA) method: measuring the spatial accessibility to hierarchical healthcare facilities in Shenzhen, China

**DOI:** 10.1186/s12939-020-01280-7

**Published:** 2020-09-21

**Authors:** Zhuolin Tao, Yang Cheng, Jixiang Liu

**Affiliations:** 1 Faculty of Geographical Science, Beijing Normal University, Beijing, 100875 China; 2 Department of Urban Planning and Design, The University of Hong Kong, Pokfulam Road, Hong Kong, China

**Keywords:** Hierarchical healthcare facilities, Spatial accessibility, 2SFCA, Absolute distance, Relative distance

## Abstract

**Background:**

Spatial accessibility to healthcare facilities has drawn much attention in health geography. In China, central and local governments have aimed to develop a well-organized hierarchical system of healthcare facilities in recent years. However, few studies have focused on the measurement of healthcare accessibility in a hierarchical service delivery system, which is crucial for the assessment and implementation of such strategies.

**Methods:**

Based on recent improvements in 2SFCA (two-step floating catchment area) method, this study aims to propose a Hierarchical 2SFCA (H2SFCA) method for measuring spatial accessibility to hierarchical facilities. The method considers the varied catchment area sizes, distance decay effects, and transport modes for facilities at various levels. Moreover, both the relative and absolute distance effects are incorporated into the accessibility measurement.

**Results:**

The method is applied and tested in a case study of hierarchical healthcare facilities in Shenzhen, China. The results reveal that the general spatial accessibility to hierarchical healthcare facilities in Shenzhen is unevenly distributed and concentrated. The disparity of general accessibility is largely caused by the concentrated distribution of tertiary hospitals. For facilities at higher levels, average accessibility of demanders is higher, but there are also larger disparities in spatial accessibility. The comparison between H2SFCA and traditional methods reveals that traditional methods underestimate the spatial disparity of accessibility, which may lead to biased suggestions for policy making.

**Conclusions:**

The results suggest that the supply of healthcare resources at primary facilities is far from sufficient. To improve the spatial equity in spatial accessibility to hierarchical healthcare facilities, various actions are needed at different levels. The proposed H2SFCA method contributes to the modelling of spatial accessibility to hierarchical healthcare facilities in China and similar environments where the referral system has not been well designed. It can also act as the foundation for developing more comprehensive measures in future studies.

## Background

Accessibility is widely used to measure the ease at which people attain opportunities or services such as healthcare, jobs, education, and retail within an area [1]. In reality, accessibility to public services usually has spatial disparities due to limited services being provided by existing public facilities. Therefore, there are usually mismatches between supply and demand of public facilities. Given their diverse residential locations, public facility users are usually faced with inequitable accessibility of public services due to the disparities in their socio-economic status and transport resources [2].

The two-step floating catchment area (2SFCA) method is among the most commonly used methods for measuring spatial accessibility [3, 4]. 2SFCA is relatively popular and advanced because it is easy to apply [1]. In recent years, a series of improvements have been developed to 2SFCA, which significantly improve the modelling of spatial accessibility [4]. Numerous studies have applied 2SFCA and its variants to measure the spatial accessibility to public services, especially healthcare [1, 4].

Hierarchical facilities refer to the facilities that function in a multi-level system, facilities at different levels provide heterogeneous services and interact with each other [5–8]. Hierarchical facilities include healthcare, education, emergency, firefighting, and logistics facilities [7, 9]. Healthcare facilities are a common type of hierarchical facility and have been widely studied in spatial accessibility measuring. Central and local governments in China have aimed to develop a well-organized hierarchical system of healthcare facilities in recent years [10–12]. The measurement of spatial accessibility to hierarchical healthcare facilities is crucial for the assessment and implementation of such strategies.

In recent decades, many researchers have made great efforts in the study of the location-allocation problem of hierarchical facilities [8, 13, 14], demonstrating the significance of considering hierarchy in facility location analysis. However, few measurements of spatial accessibility account for the hierarchical characteristics of facilities. Most existing studies examined the spatial accessibility of facilities at a single level, or analysed facilities at various levels as the same. Facilities at various levels are different in size, function, service quality, and service area [15]. If these characteristics are ignored, the assessment of spatial accessibility to hierarchical facilities would be biased and inaccurate. Therefore, it is urgent to develop novel methods for measuring spatial accessibility of hierarchical facilities. Based on the assessment of hierarchical facilities spatial accessibility, these methods can be applied to identify the service shortage areas at various levels. These questions are crucial to improve the accessibility and equity of hierarchical public services.

This study aims to develop a hierarchical two-step floating catchment area (H2SFCA) method for measuring the spatial accessibility of hierarchical healthcare facilities in China. The proposed method can reflect some key characteristics of hierarchical facilities. Drawing on an empirical dataset of hierarchical healthcare facilities in Shenzhen, China, the validity and advantages of the proposed method are tested and demonstrated. Policy suggestions for improving the provision of hierarchical healthcare services and planning of the city are also drawn.

## Literature review

### Measurements of spatial accessibility

Accessibility is a measurement of how easily and how many opportunities are accessible at different locations [16]. It not only depends on the mobility provided by transport systems but is also a function of the distribution of opportunities or destinations and individual’s socio-economic characteristics. Accessibility can be classified as spatial versus non-spatial accessibility, or potential versus revealed accessibility [17]. Spatial accessibility mainly accounts for the spatial barriers that demanders must overcome to reach services; by contrast, non-spatial accessibility focuses on socio-economic factors such as income, age, gender, and ethnicity [18]. Spatial accessibility is generally interpreted as potential accessibility, which considers the potential possibility that people access services, while revealed accessibility depends on the willingness, preference, and choices of individuals [19]. In this study, we mainly focus on spatial accessibility, which has drawn great attention in existing studies. Based on the distribution of spatial accessibility, areas or populations that face opportunity shortages can be identified, and the equity in opportunities or resources can be evaluated [19].

There are various methods for the measurement of spatial accessibility, including the nearest distance method, the kernel density method, the Huff method, the gravity method, and the cumulative opportunities method [3, 20]. Due to its understandability and operability, 2SFCA has been among the most used methods [18]. Accessibility is estimated by 2SFCA in two steps [21]. In the first step, it allocates the resources at each facility to the demand nodes within its catchment area by calculating the supply-to-demand ratios. In the second step, the supply-to-demand ratios are summed up for each demand node to calculate its accessibility.

A number of improvements have been made by recent studies based on 2SFCA [4, 22, 23], and can be classified into four types [4]. The first type of improvement modifies the distance decay functions [24–26], which can be further integrated into the generalized 2SFCA [1]. The second type of improvement strives to improve the definition of catchment areas [27–30]. The third type of improvement accounts for the impacts of the demand- or supply-side competition on accessibility [31–33]. The last type of improvement extends the assumptions concerning travel behaviors of service users [34–38]. These improvements have significantly contributed to the development and popularity of 2SFCA and even spatial accessibility modelling, but they still fail to assess hierarchical facility accessibility.

### Hierarchical facility location problem

The locations and location-allocation problem of hierarchical facilities is different from that of traditional single-level facilities. There are various types of hierarchical facilities, each of which has unique characteristics and needs different modelling techniques in location-allocation studies [7, 9]. According to a review by Şahin and Süral [7], hierarchical facilities can be classified from four dimensions, i.e. flow pattern, service varieties, spatial configuration, and optimization objective. The former three dimensions define basic characteristics of hierarchical facilities, while the last is related to the location-allocation problem.

**Fig. 1:**
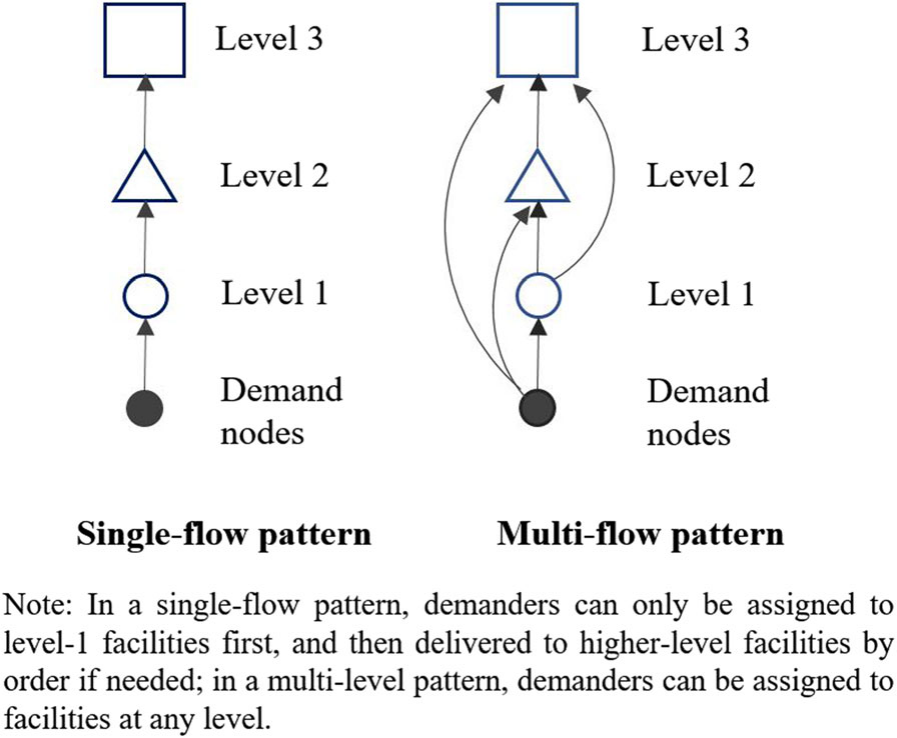
The single-flow and multi-flow pattern of hierarchical facilities

Flow pattern describes how demand for services is organized and delivered between different levels of facilities. Common flow patterns of hierarchical facilities include single-flow pattern and multi-flow pattern. As shown in Fig. 1, if a type of facility follows the single-flow pattern, the demanders at each location can only be assigned to the lowest-level facilities at the first step. Then demanders can be further transferred from the lowest-level facilities to higher-level facilities by order if higher-level services are needed. By contrast, in a multi-flow pattern, the demanders at each location can be assigned to facilities at any level. Another similar classification of hierarchical facilities is referral and non-referral facilities [39], which respectively correspond to the single-flow pattern and the multi-flow pattern.

Regarding service varieties, hierarchical facilities can be classified into nested and non-nested facilities. In a nested system, facilities at a certain level can provide services of lower-level facilities and at least one type of service that lower-level facilities cannot provide. In contrast, in a non-nested system, services provided by facilities at different levels differ from each other. As Şahin and Süral [7] pointed out, nested facilities also often have a multi-flow pattern. The classification of successively inclusive and successively exclusive facilities [40] is similar to nested and non-nested facilities.

Spatial configuration defines hierarchical facilities as spatially coherent systems or non-coherent systems. In a coherent system, demanders that are assigned to a lower-level facility must be assigned to the higher-level facility that this facility is attached to. This constraint does not apply to non-coherent systems, where demanders are assigned to a facility and have options to be assigned to multiple higher-level facilities.

Existing location-allocation models of hierarchical facilities are mainly developed based on classic location-allocation models, including the p-median model, the maximum covering model, and the least set covering problem [8, 13, 41]. As a result, the assumptions of demanders’ facility selection behaviors, i.e., interactions between demanders and facilities, in these models are quite simple. It is often assumed that demanders would choose the nearest facility (the p-median model), or demanders can be served by a facility as long as they are within a certain distance from the facility (the covering model). These modelling assumptions fail to reflect some important aspects of the accessibility across various locations to these facilities, such as competition among demanders for opportunities [42, 43]. However, there are also attempts by some researchers to incorporate spatial interaction models such as the market delineation model into hierarchical location-allocation models [14, 44, 45], though the attempts along this direction are still rare.

In this case, on one hand, efforts made by existing studies on hierarchical location-allocation problems have provided a foundation for developing methods for measuring spatial accessibility of hierarchical facilities considering their above-mentioned features. On the other hand, the development of spatial accessibility measurements for hierarchical facilities can also generate valuable feedbacks to further improve location-allocation modelling of hierarchical facilities.

### Spatial accessibility to hierarchical facilities

In recent years, partially due to the rapid development of spatial accessibility modelling, more and more researchers have begun to evaluate the spatial distribution and spatial accessibility to hierarchical facilities as well as the spatial equity effect. For instance, Song et al. analyzed the spatial distribution patterns of bi-level healthcare services and their influencing factors in China. The results show that the distribution patterns of healthcare resources are different at the two levels and are influenced by various factors [46]. Zhang et al. measured the spatial accessibility to hierarchical healthcare facilities in Chengdu, China, also revealing significant differences in spatial accessibility to healthcare services at various levels [47]. However, only the simple closest facility method, which has been considered incapable of comprehensively reflecting the concepts of spatial accessibility, was applied to measure spatial accessibility in their study [1, 22].

Several studies have strived to measure the spatial accessibility to hierarchical facilities using more comprehensive measures such as 2SFCA. Li et al. assessed the spatial accessibility to hierarchical urban parks in Shenzhen, China based on a Gaussian-based 2SFCA method. They assumed that parks at different levels might have different catchment area sizes, and that people may prefer various transport modes when traveling to such parks [48]. These two features of hierarchical facilities were then incorporated into the 2SFCA method. Similarly, Hu et al. evaluated the spatial accessibility to hierarchical healthcare services in Shenzhen, China. Specifically, two levels of healthcare services, i.e., general hospitals and community health service centers, were included. They took into account the differences in service scopes of facilities at various levels, but did not consider the availability of multiple transport modes and the variability in accessibility by using different modes [49].

Based on a 2SFCA method with power-based distance decay function, Jin et al. set up different parameters of catchment area size and distance decay for multi-level healthcare facilities. Transport modes were also treated differently according to facility levels: walking for community-level facilities and driving for district-level and municipal-level facilities. The general spatial accessibility at each demand location was then calculated based on the weighted sum of accessibility scores at each level [15]. As pointed out by Jin et al., the synthesized general accessibility of multiple levels is crucial for healthcare planning, because people have options to utilize healthcare services at any level in the Chinese healthcare system. Though their method was also termed as Hierarchical 2SFCA, it only incorporated some relatively simple characteristics of hierarchical facilities. Other key aspects of hierarchical facilities are still missing in spatial accessibility modelling, which need to be evaluated with more comprehensive methods.

Aiming to delineate hierarchical hospital service areas, Jia et al. calibrated the Huff model of patients’ hospital selection behavior using actual hospitalization records data. They found that the two-level hospital system conforms to a hierarchical central place structure. Higher-level hospitals have flatter gradients of distance decay coefficient and larger service areas [50]. These findings provide valuable empirical evidence for the assumptions made by the above studies.

Zhong et al. developed a two-stage 2SFCA method for measuring spatial accessibility to hierarchical healthcare facilities in Beijing, China, which may be the most comprehensive measurement of spatial accessibility to hierarchical facilities so far [51]. Similar to the above studies, Zhong et al’s method also reflects the variability of catchment area sizes for different levels. Moreover, their method introduces a two-stage procedure into calculation of hierarchical accessibility. At the first stage, the spatial accessibility from demand nodes to the lowest-level facilities (i.e. primary healthcare services) is estimated. The second stage deals with the referral process. It is assumed that a certain proportion of demanders need to be transferred from the lowest-level facilities to higher-level facilities for further treatment. The general accessibility is calculated as the sum of accessibility of these two stages. However, the two-stage 2SFCA method still has some shortcomings. First, it assumes that the spatial accessibility at the second stage is from lower-level facilities to higher-level facilities. In many cases, however, referral patients might not travel in this way, they may directly travel from their residential locations to the higher-level facilities. Secondly, similar to the above studies, it only considers the difference in catchment area size among facilities at different levels. The more important relative distance bias problem, which will be illustrated later, has not been recognized and addressed by existing methods for measuring spatial accessibility to hierarchical facilities.

## Case study: hierarchical healthcare system in Shenzhen, China

As shown in the literature review section, several studies have made exploratory attempts to investigate the spatial accessibility of hierarchical healthcare facilities in Shenzhen, whose results can be compared to this study to validate the proposed method. These make the hierarchal healthcare facilities in Shenzhen a proper case for this study.

### Background of Shenzhen

**Fig. 2:**
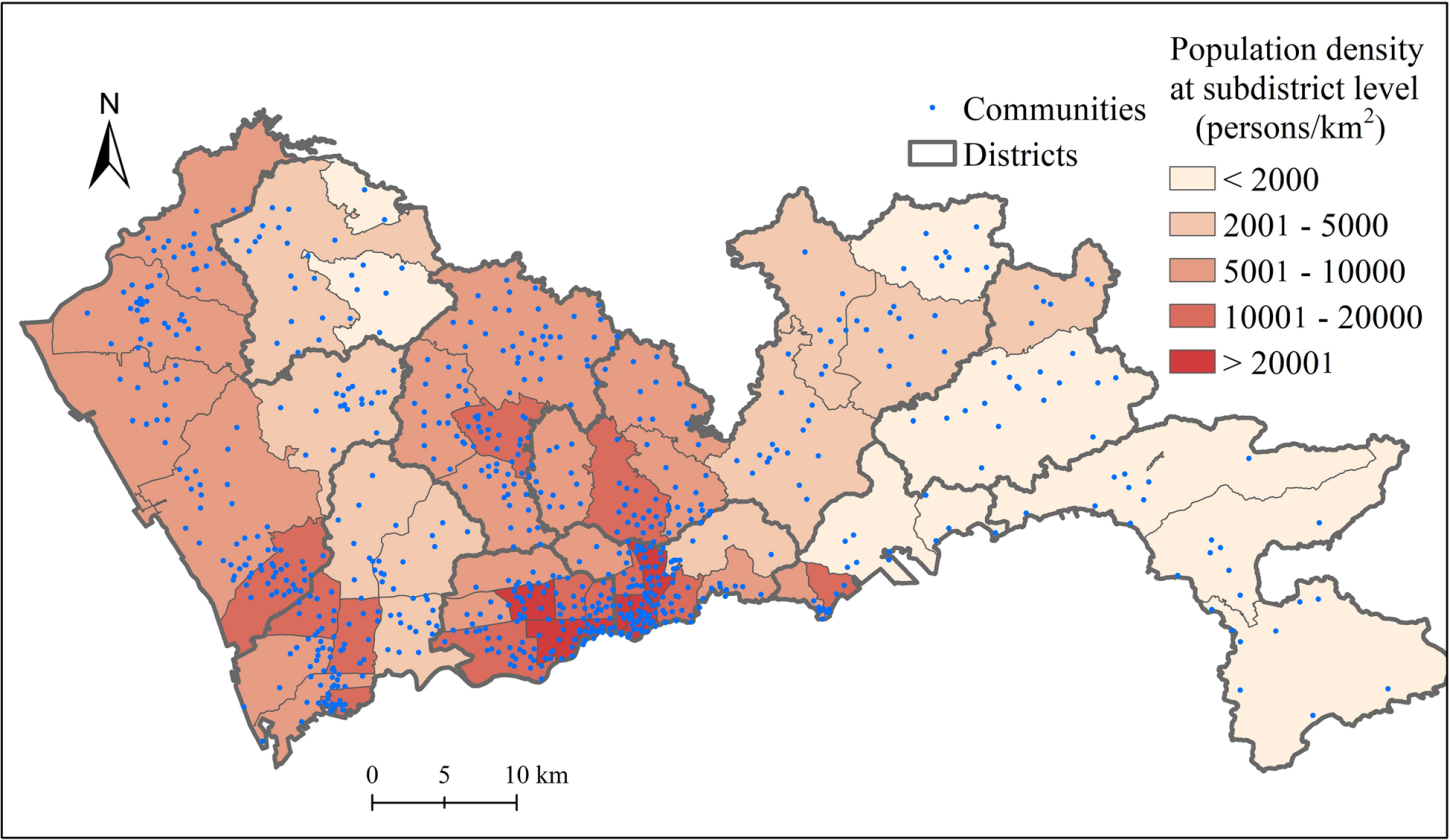
The spatial distribution of population density and communities in Shenzhen

Shenzhen, located in Guangdong Province, south China, is one of the core cities in the Pearl River Delta and one of the first-tier cities in China. After rapid growth since the reform and opening-up in the 1980s, Shenzhen has become a megacity with more than 11 million permanent residents and 1997 km^2^ of administrative area. Luohu, Futian, and Nanshan districts are the most urbanized and populous, and are usually regarded as the central city (Fig. 2).

Population data are commonly used to represent demands for healthcare services. Most existing studies, including studies on healthcare accessibility, used population data at the sub-district level for analysis. This study used the permanent population data at the community level, which is the lowest-level administrative unit in Chinese cities. These data were collected from the 6th population census data of China in 2010, which is the latest official population census data. There were 55 sub-districts and 771 communities in Shenzhen in 2010. The total permanent population was 10.35 million in 2010. On average, each community has a population of 13.4 thousand and an area of 2.6 km^2^, which is a fine resolution for studying the spatial accessibility to hierarchical healthcare facilities.

### Hierarchical healthcare system in Shenzhen, China

The data of healthcare facilities was collected from the official open data platform of Shenzhen Municipal Government (https://opendata.sz.gov.cn/) during December, 2017. This data set includes information of two types of healthcare facilities, i.e., general hospitals (GH, excluding chronic disease hospitals, occupational disease hospitals, and private hospitals) and Community Health Service Centers (CHSC). The available information includes the name, address, number of physicians, and beds of each facility. The coordinates of these facilities were collected by using Baidu Map Geocoding API (Application Programming Interface) based on their addresses. There are 54 GHs and 612 CHSCs in Shenzhen in total.

According to the “Medical Regulations of Shenzhen Special Economic Zone” [11], the healthcare service system consists of three levels of facilities. They are primary healthcare facilities (the lowest level), secondary hospitals, and tertiary hospitals in a bottom-up order. The primary healthcare facilities mainly refer to CHSCs, which are responsible for providing basic medical services such as treatments for frequently occurring diseases and chronic diseases and also recovery and nursing services. The secondary and tertiary hospitals are responsible for more skilled treatments such as emergency treatments, hospitalization, and surgeries. It is the aim to construct a hierarchical healthcare system that can be described as “primary treatments at primary facilities, two-way referral, and separate treatments of emergent and chronic diseases”. This structure is also in accord with the hierarchical healthcare system planned by the central government of China [12].

Though the central and municipal governments in China have been encouraging referral between different levels, the implementation is facing great challenges [52]. The current hierarchical healthcare system in China follows a multi-flow and nested structure. In other words, high-level facilities can provide low-level services, and patients can utilize services at any level. As a result, a large proportion of treatments are provided at high-level healthcare facilities [53]. These characteristics of the hierarchical healthcare system in China are crucial for the hierarchical spatial accessibility modelling presented in the next section.

**Fig. 3:**
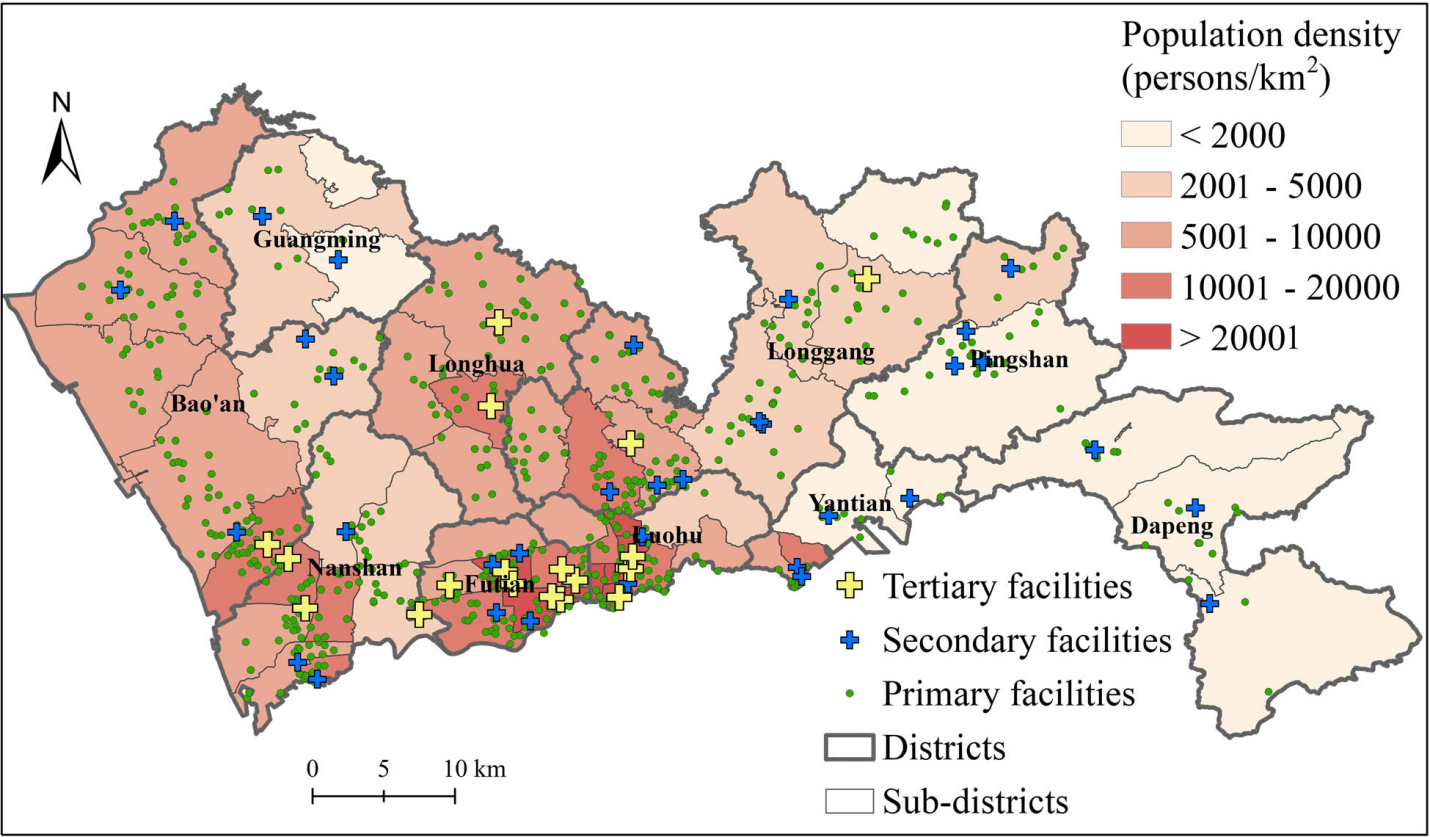
The spatial distribution of hierarchical healthcare facilities in Shenzhen

Based on the collected data, there are 612 primary facilities, 35 secondary hospitals, and 19 tertiary hospitals in Shenzhen. The average size (represented by the number of physicians) of each facility at three levels are 6, 182, and 422 physicians, respectively. As shown in Fig. 3, the spatial distribution of facilities at various levels show different patterns. The distribution of tertiary hospitals is highly uneven and concentrated, most of which are in the central districts. By contrast, secondary hospitals are distributed in most districts of Shenzhen. The only exception is Longhua district, where the two former secondary hospitals have recently been upgraded to tertiary hospitals. The primary facilities are the most numerous. There is a relative balance between the distribution of primary facilities and that of population.

## Methods

The proposed Hierarchical 2SFCA method in this study is developed based on several important advancements of 2SFCA, including the Modified 2SFCA [31], the Multiple Catchment Sizes 2SFCA [30], and the Gaussian 2SFCA [24]. The formulas of these models are uniformly expressed using the Generalized 2SFCA framework [1].

### Generalized 2SFCA

The original 2SFCA adopts a dichotomous form of distance decay function, which has been considered a major limitation. Several improved forms of 2SFCA have been developed to address this limitation. The Generalized 2SFCA proposed by Wang [1] can uniformly formulate the various forms as follows:

1 $$ {A}_i={\sum}_j\frac{S_jf\left({d}_{ij}\right)}{\sum_k{P}_kf\left({d}_{kj}\right)} $$

where *A*_*i*_ is the accessibility at location *i*, *S*_*j*_ is the size of supply of facility *j*, *P*_*k*_ is the demand size at spatial unit *k*, *d*_*ij*_ (*d*_*kj*_) is the travel cost measured by distance, travel time or total cost, and *f* is distance decay function, which can take discrete forms or continuous forms. In this study, the Gaussian function is adopted to model the distance-decay effects as suggested by existing studies [4, 24]. The Gaussian function can be formulated as:

2 $$ f\left({d}_{ij}\right)=\left\{\begin{array}{c}\frac{e^{-1/2\times {\left({d}_{ij}/{d}_0\right)}^2-}{e}^{-1/2}}{1-{e}^{-1/2}},{d}_{ij}\le {d}_0\\ {}\kern3em 0,\kern1.5em {d}_{ij}>{d}_0\end{array}\right. $$

where *d*_0_ is the size of catchment area, i.e., the threshold distance or travel time. Only one parameter is needed for the Gaussian function, making it superior to other alternatives due to less subjectivity and uncertainty caused by parameter setting.

### Relative distance Bias and M2SFCA

**Fig. 4:**
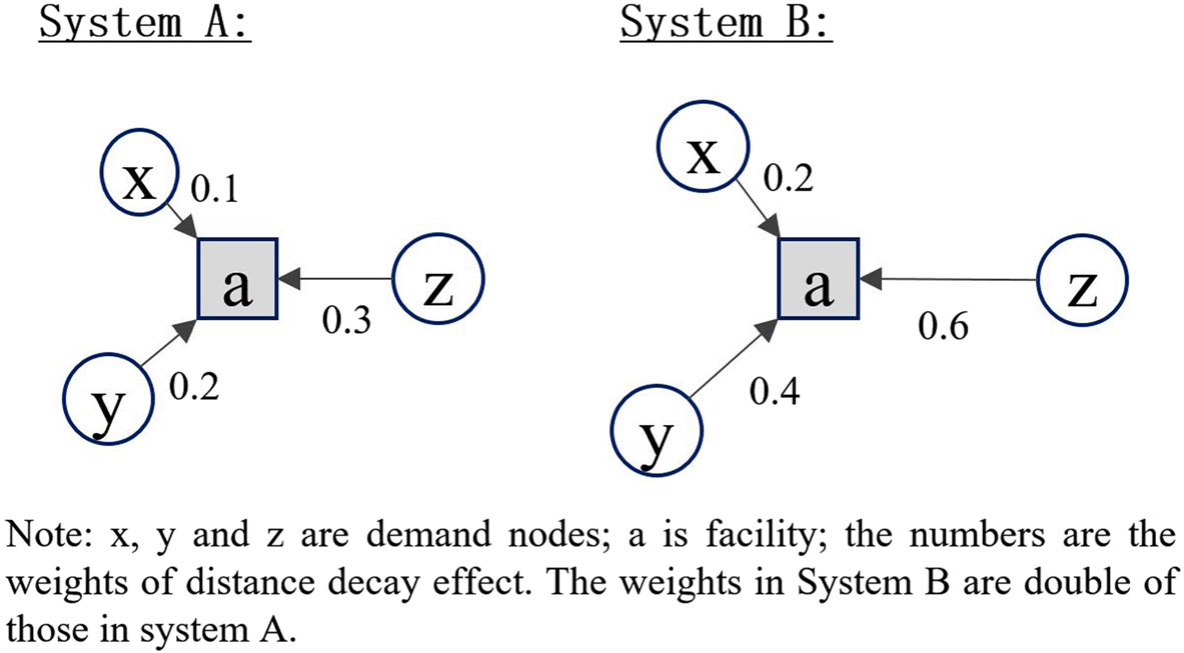
A numerical example of the relative distance bias problem of 2SFCA

The 2SFCA family of metrics, including original 2SFCA and most variants (especially those with distance decay function improvements), have a common nature: the weighted sum of accessibility scores at all locations is equal to the total supply [54]. When calculating the accessibility, the resources at each facility are allocated to the demand nodes within its catchment area based on certain weights, which are determined by the population and the relative distances from the residence locations to the facility. Delamater [31] stated that these metrics generate container-like outputs. The units of spatial accessibility estimated by these methods are the average supply (e.g., physicians) per person. This makes the results of 2SFCA metrics easy to interpret and convenient for policy making. However, this nature of 2SFCA also causes a crucial limitation, which can be explicitly demonstrated by using a simple numerical example shown in Fig. 4.

Suppose there are two different systems A and B. In each system, there is one facility (a) and three demand nodes (x, y, z). The supply of the facility and the demand sizes of three demand nodes are all set as 1. Demand nodes x, y, and z are closer to the facility in system A than in system B. In system A, the values of distance decay function (rather than the distance/travel time itself) for x, y, and z are 0.1, 0.2, and 0.3 respectively. In system B, these values are 0.2, 0.4, and 0.6 respectively, two times higher than in system A.

Based on the above settings, the spatial accessibility of each demand node in system A can be calculated as follows:

$$ {\displaystyle \begin{array}{c}\mathrm{accessibility}\ \mathrm{of}\ \mathrm{x}=1\ast 0.1/\left(1\ast 0.1+1\ast 0.2+1\ast 0.3\right)=1/6;\\ {}\mathrm{accessibility}\ \mathrm{of}\ \mathrm{y}=1\ast 0.2/\left(1\ast 0.1+1\ast 0.2+1\ast 0.3\right)=1/3;\\ {}\mathrm{accessibility}\ \mathrm{of}\ \mathrm{z}=1\ast 0.3/\left(1\ast 0.1+1\ast 0.2+1\ast 0.3\right)=1/2.\end{array}} $$

Similarly, the spatial accessibility in system B can also be calculated as follows:

$$ {\displaystyle \begin{array}{c}\mathrm{accessibility}\ \mathrm{of}\ \mathrm{x}=1\ast 0.2/\left(1\ast 0.2+1\ast 0.4+1\ast 0.6\right)=1/6;\\ {}\mathrm{accessibility}\ \mathrm{of}\ \mathrm{y}=1\ast 0.4/\left(1\ast 0.2+1\ast 0.4+1\ast 0.6\right)=1/3;\\ {}\mathrm{accessibility}\ \mathrm{of}\ \mathrm{z}=1\ast 0.6/\left(1\ast 0.2+1\ast 0.4+1\ast 0.6\right)=1/2.\end{array}} $$

As a result, the accessibility scores of three demand nodes estimated by traditional 2SFCA are the same in both systems. According to our common sense as well as the definition of spatial accessibility, the spatial accessibility in system B should be lower than that in system A, because obviously, spatial barriers are stronger in system B, while other components remain the same in both systems. However, the traditional 2SFCA methods family cannot reflect this difference. The reason is that the term of travel distance works as “relative distance” in these methods [31]. What really matters in the assessment of spatial accessibility from a demand node to a facility is the relative value of the distance from the node to the facility compared to the distances for other nodes within the catchment area of the facility, rather than the absolute value of the distance. The resulting bias can be termed as the “relative distance bias”.

Delamater [31] proposed a Modified 2SFCA (M2SFCA) method which incorporates an additional term of absolute distance into the 2SFCA method. By doing so, spatial accessibility will depend on both relative distance and absolute distance between demand nodes and facilities. According to M2SFCA, the spatial accessibility in system B is lower than that in system A as expected.

M2SFCA can be expressed as:

3 $$ {A}_i=\sum j\frac{S_jf\left({d}_{ij}\right)f\left({d}_{ij}\right)}{\sum_k{P}_kf\left({d}_{kj}\right)} $$

where the meanings of all variables are the same as Eq. (1). In Eq. (3), the absolute distance is represented by *f*(*d*_*ij*_). Given that *f* is a monotone decreasing function, a larger distance *d*_*ij*_ indicates a smaller value of *f*, and thus lower accessibility. The term $\frac{f\left({d}_{ij}\right)}{\sum_k{P}_kf\left({d}_{kj}\right)}$ describes the impact of relative distance on accessibility. The numerator denotes the absolute distance effect for demand node *i*, and the denominator denotes the sum of absolute distance effects for all nodes within the catchment area of facility *j*, which are weighted by the amount of demand. Therefore, the whole term indicates the relative value of a node’s distance compared to the distances of other nodes.

### Relative distance Bias in hierarchical systems

Delamater [31] explained that it is assumed the facility system is optimally configured in traditional 2SFCA methods, whereas in reality, the system configuration is sub-optimal in most situations. The only situation where the configuration of facility system is optimal is when all demand nodes are overlapped with facilities, i.e., the distances from demand nodes to facilities are zero. In a sub-optimally configured system, resources of facilities cannot be fully exploited. The larger the separation between demand nodes and facilities, the lower the exploitation rate of resources.

**Fig. 5:**
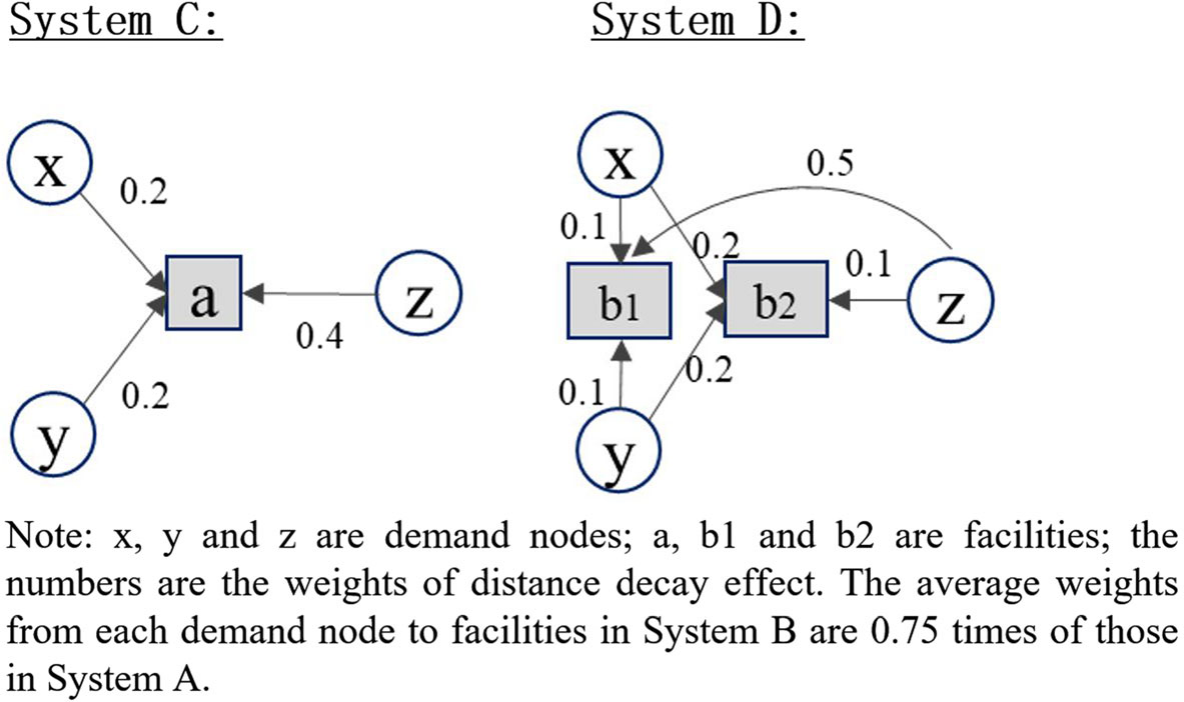
A numerical example of the relative distance bias in a bi-level system

However, Delamater [31] only considered the situation of single-level facilities and attributed the relative distance bias to sub-optimal configuration of facility systems. It will be shown in this study that the relative distance bias also exists in a hierarchical facility system, albeit in a different way. This can also be demonstrated using a numerical example shown in Fig. 5.

This example deals with a bi-level system. In system C, there is one level-2 (the higher level) facility (a) whose supply size is 8. In system D, there are two level-1 facilities (b1, b2) whose supply sizes are both 4. Both systems have three demand nodes (x, y, z) with their demand size as 1. In system C, the values of distance decay function are 0.2, 0.2, and 0.4 for demand nodes x, y, and z, respectively. In system D, these values are 0.1, 0.1, and 0.5 for x, y, and z for facility b1, and 0.2, 0.2, and 0.1 for x, y, and z for facility b2, respectively. According to traditional 2SFCA methods, accessibility in both systems would be the same, with the accessibility of x, y, and z being 2, 2, and 4 respectively. However, in system C, the spatial barriers from x, y, and z to facility a are 0.2, 0.2, and 0.4 respectively. In system D, the average spatial barriers from three nodes to the two facilities are 0.15, 0.15, and 0.3 respectively. That is to say, in system D demanders are closer to facilities on average than in system C. The spatial accessibility in system D should be higher than that in system C with absolute distance effect taken into account.

The above example explicitly reveals the existence of relative distance bias in hierarchical systems. This is embedded in the allocation of resources among different levels of facilities. In a hierarchical system, higher-level facilities often have larger sizes but a smaller number of facilities, and therefore are on average more distant from demanders that are sparsely distributed across the space. As a result, given a certain amount of resources, i.e., physicians in healthcare facilities, if these resources are configured at a lower level, they are supposed to be closer to demanders on average and therefore generate higher accessibility. In a hierarchical system, both the relative distance and absolute distance effects should be considered in measuring spatial accessibility to eliminate the relative distance bias generated by traditional 2SFCA methods. M2SFCA can be utilized to achieve this goal and to generate better estimation of the spatial accessibility to hierarchical facilities.

### Multiple catchment sizes 2SFCA

Another important feature of hierarchical facilities is that facilities at various levels often have different service scopes. Generally, higher-level facilities serve demanders within larger service areas. The variable catchment areas should be incorporated into the measurements of spatial accessibility to hierarchical facilities. Tao et al. [30] developed a Multiple Catchment Sizes 2SFCA method (MC2SFCA), which determines catchment area sizes according to facilities’ sizes. They supposed that larger facilities generally have larger catchment areas that provide services for more demanders. The method was validated by using a case of residential care facilities in Beijing.

This study proposes to modify the MC2SFCA method for measuring the spatial accessibility to hierarchical facilities. When applied to a hierarchical system, the catchment area sizes are determined based on the hierarchies of facilities, rather than their sizes as in the original form. Higher-level facilities should be assigned with larger catchment areas. Furthermore, though no distance decay parameter is specified in Gaussian-based 2SFCA models, the catchment size parameter (*d*_0_ in Eq. (2)) also determines the slope of distance decay function. A larger catchment size means a weaker distance decay effect. In this sense, the distance decay function is flatter for higher-level facilities. These assumptions are in accord with the central place theory and has been verified by the empirical analysis of Jia et al. [50].

### Hierarchical 2SFCA

The proposed Hierarchical 2SFCA (H2SFCA) method is developed by combining Gaussian-based 2SFCA, M2SFCA, and MC2SFCA, which have been described above. These methods are redefined in hierarchical systems, to reflect several important features of hierarchical facilities. The proposed H2SFCA method can be expressed as:

4 $$ {HA}_i={\sum}_l{\sum}_{j\in \left\{{d}_{ij}\le {D}^l\right\}}\frac{S_j^lf\left({d}_{ij}\right)f\left({d}_{ij}\right)}{\sum_{k\in \left\{{d}_{kj}\le {D}^l\right\}}{P}_kf\left({d}_{ij}\right)} $$

where *HA*_*i*_ is the general spatial accessibility at location *i* in a hierarchical system, *l* is the level of facilities in the system, ${S}_j^l$ is the supply of facility *j* at level *l*, *D*^*l*^ is the travel time threshold (i.e. catchment area size) for facilities at level *l*. The distance decay function *f* takes a Gaussian function form as follows:

5 $$ f\left({d}_{ij}\right)=\left\{\begin{array}{c}\frac{e^{-1/2\times {\left({d}_{ij}/{D}^l\right)}^2-}{e}^{-1/2}}{1-{e}^{-1/2}},{d}_{ij}\le {D}^l\\ {}\kern4em 0,\kern1.25em {d}_{ij}>{D}^l\end{array}\right. $$

where all variables are the same as Eq. (4).

### Estimation of travel time and catchment sizes

This study uses the Driving Searching API provided by Baidu Map to estimate travel time from demand locations to hierarchical facilities. API allows researchers to obtain reliable estimations of travel time by using the data provided by map service providers [55]. Descriptions of Baidu Map API can be found in previous studies [37, 56]. The estimation of travel time is between 10 a.m. and 5 p.m. on work days to avoid the impacts of traffic congestion during rush hour, which is more related with commuting travel rather than travel to hospitals.

Different transport modes should be considered when measuring the travel costs to facilities at different levels in a hierarchical system. In general, most patients travel relatively long distances to reach tertiary and secondary hospitals by using motorized transport. Therefore, the travel times from demand locations to tertiary and secondary hospitals were estimated using the above API approach. Primary facilities (mainly CHSCs) are roughly configured at the community level and are relatively close to patients’ residential locations. The mean distance from each community to the closest primary facility is 0.55 km. 96.4% communities (743 out of 771) are located less than 2 km from the closest primary facility. Therefore, non-motorized transport such as walking and cycling is preferred for visiting primary facilities. In recent years, due to the “boom of shared bikes” such as Mobike and OFO, cycling has become popular again for daily trips in Chinese cities. We consider cycling as the main transport mode for trips to primary facilities. According to a recent survey report [57], the average speed of shared bike riders in Chinese cities is 9 km/h. The distances from demand nodes to primary facilities are estimated based on actual road networks by using ArcGIS 10.5.

The proposed H2SFCA also assumes that facilities at various levels have different service areas (i.e., catchment areas) in a hierarchical system. Referencing existing studies [37, 56], the catchment area size was set as the maximum travel time by public transport from each demand node to the closest facility, so that all demanders at each location can access at least one healthcare facility within the catchment area. By doing so, the catchment area sizes *D*^*l*^ are set as 25 min, 40 min, and 70 min for facilities at three levels. Based on the above data and settings, the spatial accessibility at each demand location (i.e., communities) to each level of healthcare facilities, as well as the general accessibility, can be estimated. Since the outputs were accessibility scores at discrete locations, the spatial interpolation analysis is further conducted to generate continuous surface of spatial accessibility distribution. Specifically, the Inverse Distance Weighted method provided in ArcGIS 10.5 was applied to do interpolation.

## Results

### General spatial accessibility to hierarchical healthcare facilities by H2SFCA

**Fig. 6:**
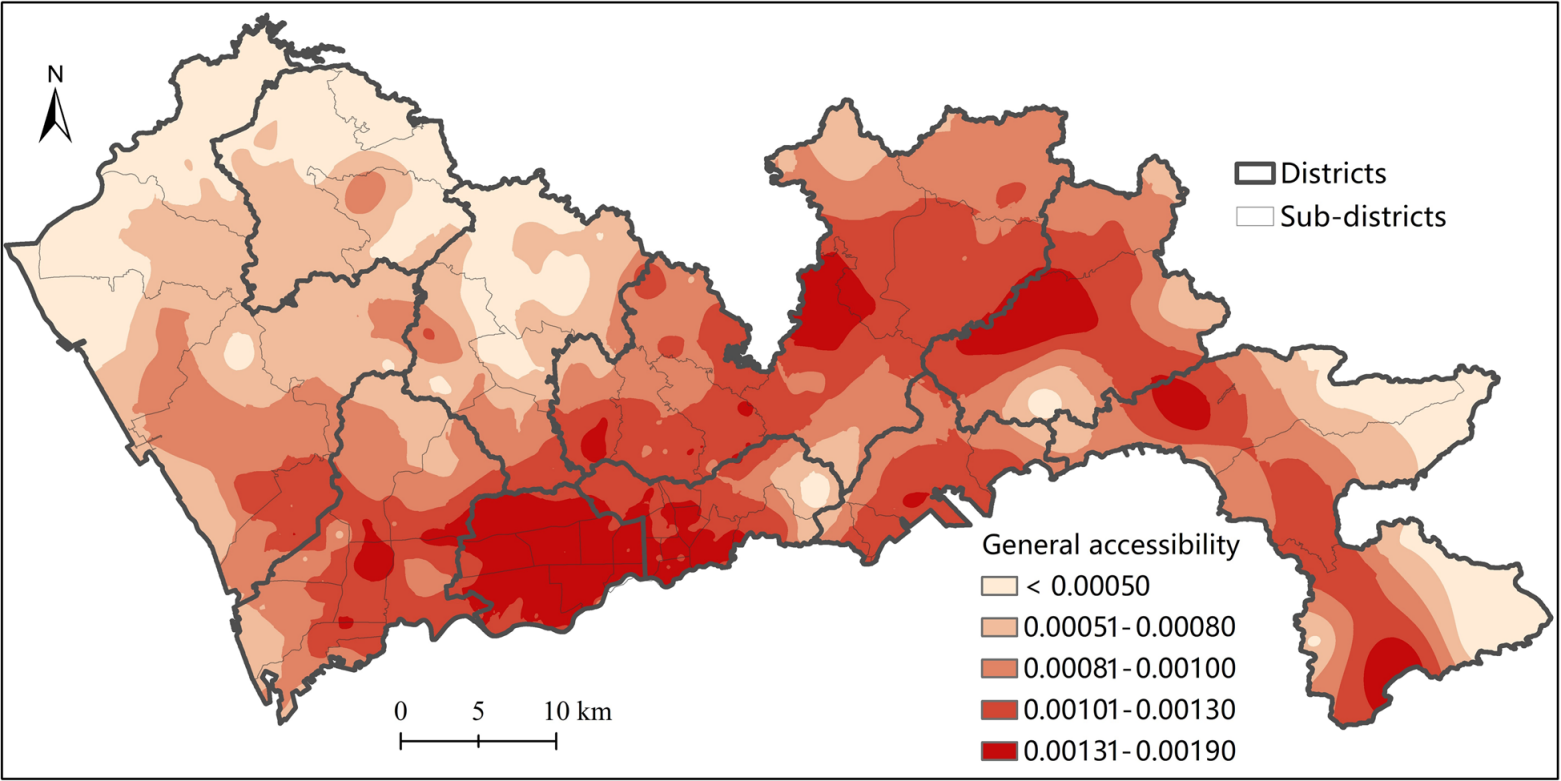
General accessibility to hierarchical healthcare facilities by H2SFCA in Shenzhen

The general spatial accessibility calculated by Eq. (4) is a comprehensive measurement of the spatial accessibility to healthcare facilities at all levels. As shown in Fig. 6, the general spatial accessibility to hierarchical healthcare facilities in Shenzhen is highly unevenly distributed. The highest general accessibility is concentrated in Futian and Luohu Districts, which are the main central districts of Shenzhen. Futian and Luohu Districts also have the highest population density and the most concentrated healthcare facilities, especially higher-level facilities. Considering that the spatial accessibility index takes both demand and supply into account, the uneven distribution of accessibility reveals significant mismatch between the supply and demand of healthcare services. This reveals that the distribution of healthcare resources is even more concentrated than that of population. Notably, among the 19 tertiary hospitals, 12 are located in Futian and Luohu Districts. In addition to Futian and Luohu, the general accessibility in the eastern part of Shenzhen, including Longgang, Pinghan, Yantian, and Dapeng Districts, is generally higher than that of the western part, including Nanshan, Longhua, Baoan, and Guangming Districts.

### Healthcare spatial accessibility at each level by H2SFCA

The healthcare spatial accessibility at each level can be further visualized. As the results show, the healthcare spatial accessibility at various levels significantly differ from one another.

The population-weighted average healthcare accessibility at each level is calculated by multiplying the accessibility at each location by the population and then summing them up, which is shown in Table 1. Though the amounts of facilities are much fewer at higher levels, the average accessibility is notably higher at higher levels. The gap is the largest between the accessibility to primary facilities (level 1) and the accessibility to secondary and tertiary hospitals (level 2 and 3). Considering that the demand is the same at each level, the average accessibility at each level is determined by either the supply sizes or the distance from facilities to demanders on average. Table 1 also gives the total supply of healthcare resources (measured by number of physicians) at each level. The total supply at level 2 is 1.73 times that at level 1, while level 3 is 1.26 times that at level 2. By contrast, the average accessibility at level 2 is 1.49 times that at level 1, and the average accessibility at level 3 is 1.16 times that at level 2. The inter-level difference in accessibility is less than that in total supply. This suggests that the closer distance from lower-level facilities to demanders on average gives them some advantages in generating healthcare accessibility.

**Table 1:** The weighted average healthcare accessibility and disparity at different levels

Levels	Level 1	Level 2	Level 3
Weighted average accessibility	2.26	3.37	3.92
Total supply	3672	6370	8018
Standard deviation (multiplying 10^4^)	0.79	1.6	3.2
Coefficient of variation (CV)	0.35	0.48	0.54

In a word, the above results reveal the unbalanced configuration among different levels in the hierarchical healthcare system in Shenzhen. More resources are configured at higher levels, resulting in higher average accessibility at higher levels. Facilities at lower levels are closer to demanders on average, and therefore can improve the accessibility at lower levels, but only to a limited extent.

**Fig. 7:**
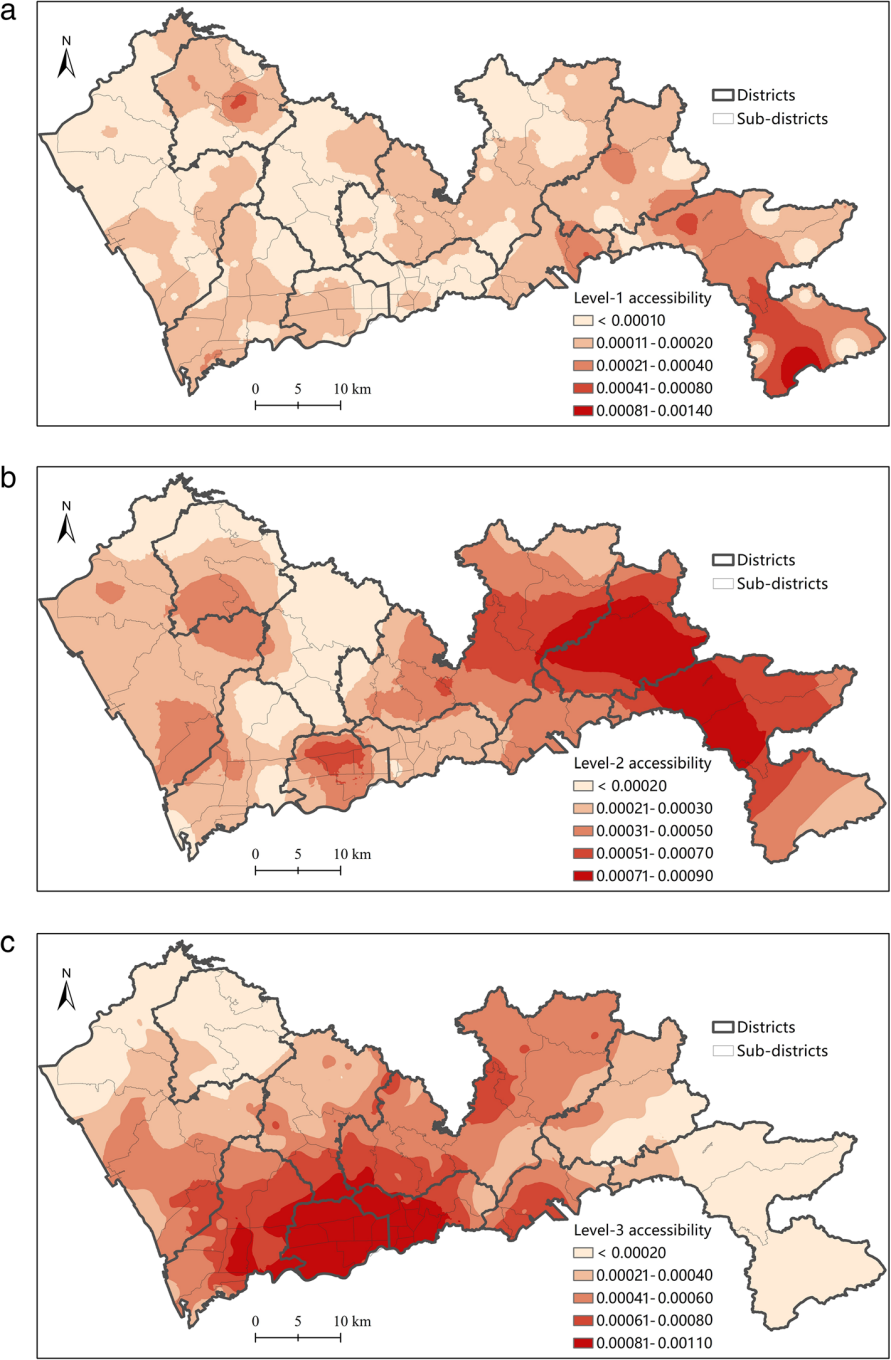
Spatial accessibility to (**a**) primary; **b** secondary; **c** tertiary facilities by H2SFCA in Shenzhen

As shown in Fig. 7, the distributions of accessibility at the three levels are also different. The accessibility to tertiary hospitals is the most unevenly distributed, which presents an obvious monocentric pattern. Futian and Luohu Districts has the highest accessibility to tertiary hospitals. This is roughly in accord with the distribution of tertiary hospitals.

The distribution of the accessibility to secondary hospitals is quite different with that of tertiary hospitals. Futian and Luohu no longer have the highest healthcare accessibility at this level. This is because Futian and Luohu have the highest population density, while the distribution of secondary hospitals is dispersed and relatively even across most of the districts. By contrast, the areas in Pingshan, Longgang, and Dapeng Districts in eastern Shenzhen have the highest accessibility to secondary hospitals. The accessibility to secondary hospitals is the lowest in Longhua district and its surrounding areas. Longhua is the only district that has no secondary hospitals. This is because the former secondary hospitals in Longhua have been upgraded to tertiary hospitals, but no new secondary hospitals have been built so far.

The distribution of the accessibility to primary facilities is the most even among the three levels. The highest accessibility only appears in a few areas in Dapeng, Yantian, and Pingshan Districts. This does not imply that supply of primary facilities is abundant in these areas. In contrast, this is because population density is quite low in these areas. The accessibility to primary facilities is relatively low in most areas in Shenzhen.

Based on the analysis of accessibility at each level, it can be observed that the spatial difference in accessibility to tertiary hospitals seems to mostly contribute to the disparities of general accessibility followed by the disparities of secondary hospital accessibility, while the disparity of primary healthcare accessibility contributes the least. The standard deviation statistics (in Table 1) further support this observation. The standard deviation, which can roughly reflect their contributions to the differences in general accessibility, is larger at higher levels facilities. By contrast, the coefficient of variation (CV) reflects the relative variation of variables, with the scale of variables taken into account. Similarly, CV of healthcare accessibility is larger at higher levels in the hierarchical system. This also indicates that the disparity of accessibility is the largest for tertiary hospitals and least for primary facilities.

### Comparison with traditional 2SFCA method

In this section, a comparison is made between the results of the proposed H2SFCA and that of the traditional 2SFCA. Note that the traditional 2SFCA method used in this study also incorporates a Gaussian distance decay function. The differences between two methods is whether the relative distance bias is addressed: H2SFCA takes into account both relative distance and absolute distance effects, while traditional 2SFCA only considers relative distance effect.

**Fig. 8:**
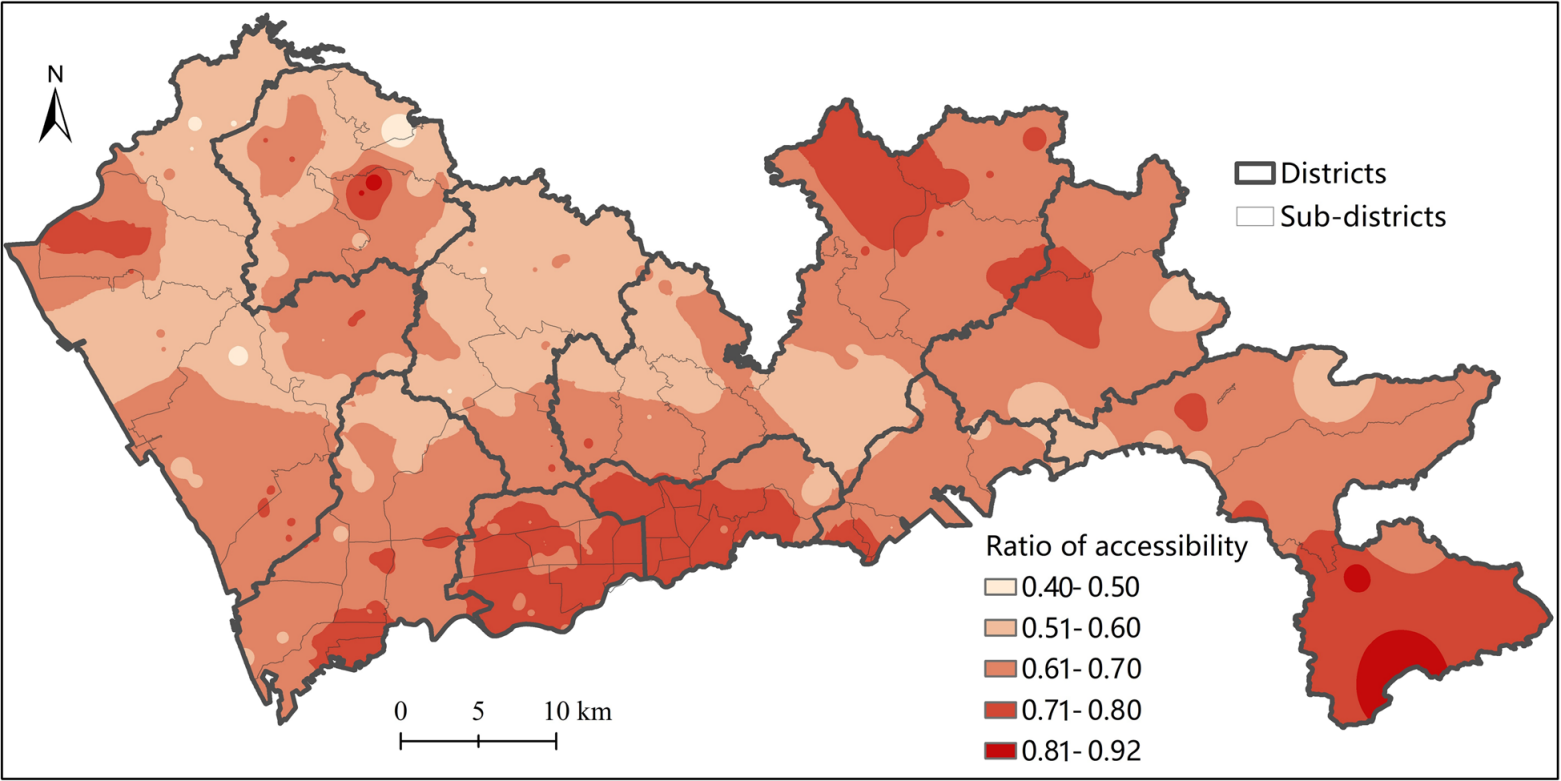
Ratio of accessibility by H2SFCA to accessibility by traditional 2SFCA

Figure 8 visualizes the ratio of general accessibility calculated by H2SFCA to that by traditional 2SFCA. The ratio is less than 0.92 for all locations, indicating that the accessibility by H2SFCA is lower than that of traditional 2SFCA. This is because, compared to traditional 2SFCA, an additional distance decay function is incorporated into H2SFCA to model the absolute distance effect. The value of distance decay function ranges between 0 and 1.

What matters more is the distribution of this ratio of accessibility. It follows a relatively concentrated pattern, which is similar to the distribution of the general accessibility by H2SFCA. That is to say, for areas with a lower general accessibility, the difference between the accessibility by H2SFCA and that by traditional 2SFCA tends to be larger. Therefore, the disparity of accessibility by H2SFCA is larger than that by traditional 2SFCA. For a hierarchical system, the traditional spatial accessibility methods would underestimate the disparities of accessibility.

## Discussion

The discussion is mainly twofold, with one about the empirical findings and implications for policy making in Shenzhen, and the other about the modelling of spatial accessibility of hierarchical systems.

The results reveal that the general spatial accessibility to hierarchical healthcare facilities in Shenzhen is unevenly distributed and concentrated. The accessibility in the central districts (Luohu and Futian Districts) is notably higher than that in peripheral districts, and the accessibility in eastern Shenzhen is higher than that in western Shenzhen. Moreover, the disparity of general accessibility is largely caused by the concentrated distribution of tertiary hospitals. For facilities at higher levels, average accessibility of demanders is higher, but there are also larger disparities in spatial accessibility.

These results suggest that the supply of healthcare resources at primary facilities is far from sufficient. Though the amount of primary facilities is much larger than secondary and tertiary hospitals, the average size of primary facilities is quite small. To improve the spatial equity in the spatial accessibility to hierarchical healthcare facilities various actions are needed for different levels of facilities. For tertiary hospitals, building new hospitals in the periphery districts can improve spatial accessibility. For secondary hospitals, more services can be provided in Longhua District and the surrounding areas to improve the lowest accessibility to secondary hospitals under the current pattern. Primary facilities can serve most of the areas in the current state, however, more effort should be made to expand existing facilities rather than to plan new ones. By doing so, the capacity of each primary facility to provide primary healthcare services can be improved.

As a leading city in the reform and opening up in China, Shenzhen’s economic level is among the best in the country and the health insurance coverage rate has reached 100%. The health insurance system in Shenzhen is divided into three levels with various contribution and reimbursement rates, namely comprehensive health insurance, hospitalization health insurance, and health insurance for migrant workers. The ones who are enrolled in level 1 health insurance can access any designated health care facility in the city. Although those who are enrolled in the other two levels of health insurance can only choose a primary health care facility for outpatient visits, the selected facility can be changed in the following month if they are not satisfied. In 2020, 34.7% residents were enrolled in level 1 health insurance, while 43.5 and 21.8% residents were enrolled in level 2 and 3 health insurance, respectively. Health insurance reform helps to improve the financial aspect and the equity in access to health care services among residents in Shenzhen [58].

By simultaneously incorporating the relative and absolute distance effects, H2SFCA is capable of reflecting the advantage of lower-level facilities due to closer distance to demanders, generating higher accessibility. This property of H2SFCA has been verified based on the empirical results. According to H2SFCA, the average accessibility at each level depends on the total supply and the distance from demanders to facilities on average. In our case study, the total supply at the lower level is less, but the differences in accessibility between various levels is less than the differences in total supply. This confirms that the closer distance from lower-level facilities to demanders can improve their accessibility to a certain extent. In a word, for a certain amount of healthcare resources, configuring them at a lower level in the hierarchical system can generate larger spatial accessibility benefits.

Some studies have evaluated the spatial accessibility to hierarchical healthcare facilities in Shenzhen or other similar contexts. Hu et al. [49] measured the spatial accessibility to the bi-level healthcare facilities in Shenzhen. All GHs were considered as one level. The results revealed a similar pattern that healthcare accessibility to GHs is more concentrated, while accessibility to CHSCs is more dispersedly and evenly distributed. In their study, only the original 2SFCA and gravity model were utilized. In this sense, they incorporated different catchment sizes and distance decay effects into the modelling of spatial accessibility of a hierarchical system. However, the distance decay trend was set as flatter for CHSCs, which is against the rule verified by Jia et al. [50] and the settings in other studies.

Jin et al. [15] also evaluated the spatial accessibility to hierarchical healthcare facilities in Shenzhen. They used more appropriate settings of hierarchical healthcare facilities, including larger catchment sizes and flatter distance decay functions for higher-level facilities. They also used the same data set as this study. In their results, the spatial accessibility patterns at three levels are quite similar to our study. However, Jin et al. did not address the relative distance bias in the hierarchical system. According to our analysis and comparison, this would underestimate the spatial disparity of general accessibility. The differences in the distribution of general accessibility between their study and ours can also clearly verify this underestimation.

The modelling of the spatial accessibility to hierarchical facilities (e.g., healthcare facilities) is a field that is still under exploration. The proposed H2SFCA method in this study reflects the characteristics of hierarchical systems in four aspects. First, the catchment area sizes are set as different for facilities at various levels. Facilities at higher levels have larger catchment areas. Second, the distance decay effect on spatial accessibility is weaker for higher-level facilities. This is determined by the size of catchment area in the Gaussian-based distance decay function. Third, the transport modes are different for trips to facilities at various levels. Lastly and most importantly, both the relative and absolute distance effects are incorporated into the accessibility measurement. The relative distance effect is modeled in traditional 2SFCA methods, including the original 2SFCA and most variants.

It is worth noting that the method proposed by Jin et al. [15] was also termed as Hierarchical 2SFCA. Their version of H2SFCA incorporates the first three of the above mentioned four characteristics of hierarchical systems. However, the method fails to take the absolute distance effect into account, which is crucial for modelling the spatial accessibility to hierarchical facilities.

Due to the incorporation of absolute distance effect, the H2SFCA proposed in this study can reflect the potentials of lower-level facilities in improving spatial accessibility. This is because lower-level facilities are closer to demanders on average due to their large numbers and more dispersed distribution. This property of H2SFCA is helpful to the evaluation of different policies regarding the configuration of resources at various levels in a hierarchical system. What matters in shaping the pattern of spatial accessibility not only depends on the spatial distribution of facilities, but also how the resources are allocated at various levels of these facilities.

It should not be concluded, however, that allocating more resources at lower-level facilities is always feasible and beneficial. In fact, despite the above characteristics, there are some other factors that should be considered in the configuration of hierarchical facilities. Among these, the specialized functions of each level and economies of scale in the provision of services might be of great importance. Future work needs to be done to identify and quantify these factors.

Nevertheless, the H2SFCA proposed by this study has made contributions in modelling the spatial accessibility to hierarchical facilities. It should also be noted that the proposed H2SFCA method is only applicable for multi-flow and nested hierarchical systems. It does not account for the referral between facilities at different levels. The current hierarchical healthcare system in China meets these conditions. It should be applicable in the context of other developing countries where the referral system has not been well developed. Furthermore, H2SFCA can act as the foundation of more comprehensive models in the future.

In addition, it should be kept in mind that spatial accessibility is important but only one of multiple dimensions of the accessibility to healthcare services. The planning and investment of healthcare facilities should comprehensively account for spatial accessibility as well as non-spatial accessibility to healthcare facilities. For example, non-spatial accessibility may refer to financial, social, and ethnic barriers to healthcare services.

## Conclusions

Spatial accessibility to public service facilities, such as healthcare facilities, has drawn much attention in recent years. However, few have strived to evaluate the spatial accessibility to hierarchical facilities, due to the lack of comprehensive measurement. Indeed, most of the facilities that have been widely evaluated in studies on spatial accessibility are hierarchical.

Based on the analysis of basic characteristics of hierarchical facilities, this study has proposed a Hierarchical 2SFCA method for measuring the spatial accessibility to hierarchical facilities. This method was developed by incorporating and modifying several recent advancements of the widely used 2SFCA method to reflect the characteristics of hierarchical facilities. The method can quantify the spatial accessibility to hierarchical healthcare services as well as its differences at various levels. The method has been applied in a case study of hierarchical healthcare facilities in Shenzhen, China. Policy suggestions on potential improvements of the hierarchical healthcare system in Shenzhen have also been drawn based on the empirical findings. These suggestions can also provide reference for other Chinese cities where the mechanism of hierarchical healthcare system is similar to Shenzhen. The proposed H2SFCA method can act as the foundation for development of more comprehensive hierarchical spatial accessibility measurements in future studies.

This study also has some limitations. First, public transport such as metro and bus are not included in this study. In fact, the Baidu Map API introduced in this study can also be exploited to estimate travel time by public transport but it is highly time-consuming, and thus is not applied in this study. Second, some important advancements in spatial accessibility measurement are not applied in this study, such as the multi-modal 2SFCA method. Third, other characteristics of hierarchical facilities are still excluded from the method, such as the specialized functions of each level and economies of scale that have been discussed in the former section. Analyses based on detailed data of actual utilization behaviors of hierarchical facilities can also help develop more comprehensive methods in the future. Lastly, this study mainly focuses on spatial accessibility and spatial equity. More dimensions of healthcare accessibility (e.g., financial accessibility, socio-cultural accessibility) should be comprehensively investigated in future studies.

## Data Availability

The datasets used and/or analysed during the current study are available from the corresponding author on reasonable request.

## Abbreviations

2SFCA: Two-step floating catchment area
API: Application programming interface
CHSC: Community health service centers
CV: Coefficient of variation
GH: General hospitals
H2SFCA: Hierarchical 2SFCA
M2SFCA: Modified 2SFCA
MC2SFCA: Multiple catchment sizes 2SFCA
